# Change and relationship between growing season metrics and net primary productivity in forestland and grassland in China

**DOI:** 10.1186/s13021-023-00245-x

**Published:** 2023-12-21

**Authors:** Linli Cui, Jun Shi, Fengjin Xiao

**Affiliations:** 1 Shanghai Ecological Forecasting and Remote Sensing Center, Shanghai Meteorological Bureau, Shanghai, 200030 China; 2 Qingpu Meteorological Station of Shanghai, Shanghai, 201700 China; 3 National Climate Center, China Meteorological Administration, Beijing, 100081 China

**Keywords:** Growing season metrics, Net primary productivity, Forestland and grassland, Spatial trend, Inter-decadal variation, Different regions of China

## Abstract

**Background:**

Vegetation phenology can characterize ecosystem functions and plays a key role in the dynamics of plant productivity. Here we investigated the changes in growing season metrics (start of growing season, SOS; end of growing season, EOS; length of growing season, LOS) and their relationships with net primary productivity (NPP) in forestland and grassland in China during 1981–2016.

**Results:**

SOS advanced, EOS delayed, LOS prolonged and NPP increased significantly in 23.7%, 21.0%, 40.5% and 19.9% of the study areas, with an average rate of 3.9 days decade^−1^, 3.3 days·decade^−1^, 6.7 days·decade^−1^ and 10.7 gC m^−2^·decade^−1^, respectively. The changes in growing season metrics were obvious in Northwest China (NWC) and North China (NC), but the least in Northeast China (NEC). NPP was negatively correlated with SOS and positively correlated with EOS and LOS in 22.0%, 16.3% and 22.8% of the study areas, respectively, and the correlation between NPP and growing season metrics was strong in NWC, NC and Southwest China (SWC), but weak in NEC and South China (SC).

**Conclusion:**

The advanced SOS, delayed EOS and prolonged LOS all contribute to the increased NPP in forestland and grassland in China, especially in NWC, NC and SWC. This study also highlights the need to further study the response of NPP to growing season changes in different regions and under the influence of multiple factors.

## Background

Over the past century, the Earth’s climate has experienced major changes characterized primarily by global warming, and global surface temperature is 0.99 °C higher in 2001–2020 than in 1850–1900 [1]. Climate change is of great concern in many parts of the world because of its direct and indirect impacts on the economy, human health and ecosystems [1, 2]. Vegetation phenology and net primary productivity (NPP) are important indicators of carbon storage and carbon cycle in terrestrial ecosystems, and play a vital role in global climate change and biogeochemical cycles [3–5]. An increasing number of studies have analyzed the influence of climate factors such as temperature and precipitation on phenology and NPP, especially the asymmetric effects of nighttime and daytime warming [4–8]. In recent decades, climate change has led to an advanced start of the growing season (SOS), a delayed end of the growing season (EOS) and a prolonged length of the growing season (LOS) in most of the Northern Hemisphere [9–13], which has altered the physiological and ecological processes in plants and has obvious impacts on NPP [14–18]. Quantifying the changes in growing season and NPP and their relationships is of great significance for assessing the effects of global warming on ecosystem carbon patterns and predicting future ecosystem carbon dynamics [9, 18].

Located in the southeast of Eurasia, most of China belongs to the East Asian monsoon climate zone [19]. The advance and retreat of monsoon and corresponding rainfall have significant effects on the growing season dynamics and annual NPP. Some studies have investigated the changes in NPP and their relationship to the starting, ending and length of the growing season, for example, Liu et al. [20] analyzed the spatial and temporal variation of vegetation NPP in China and showed that NPP had a fluctuating increase trend during 2001–2014. Ma et al. [4] explored the variation of NPP in temperate grasslands in China and its response to climate change from 2000 to 2020. Qiu et al. [17] examined the variation of NPP in Northeast China and found that NPP increased in most forestlands but decreased in grasslands, meadows and forest-agricultural ecotones during 1982–2013. Shen et al. [5] analyzed the variation of NPP in marshes of the Qinghai-Tibet Plateau and its relationship with climate factors. Dong et al. [15] found that annual NPP was positively correlated with LOS in desert steppe in Inner Mongolia, and the increase of NPP was more closely related to the delay of EOS in autumn. Yang et al. [21] showed that the extension of LOS had led to the increase of NPP in the Qinghai-Tibetan Plateau, and the advanced SOS had a greater impact on NPP than the delayed EOS.

However, as far as we know, most of the existing studies have used remote sensing or model data from different sources to investigate the regional changes in growing season [6, 8, 12, 22], NPP [4, 23] or the relationships between growing season metrics and NPP in parts of China [14–18, 21, 24], and few studies used uniform data to study national NPP [7, 20], growing season metrics [11] and their relationships and regional differences across the entire China. Whether and how the growing season patterns affect NPP across China remains unclear. Moreover, forests and grasslands, as the most important natural vegetation in China, have made remarkable achievements in national carbon storage and carbon sink in recent decades [7, 25], but the relationship between NPP and growing season in forestland and grassland remains controversial. For example, in the northern Tibetan Plateau with desert steppe, alpine meadow, shrubs, marshland, and desert ecosystems, both SOS and EOS were negatively correlated with NPP [16], but in the desert steppe of Inner Mongolia, SOS was non-significantly correlated with spring NPP, and EOS was positively correlated with autumn NPP [15]. Therefore, the aim of the present study is to investigate the changes in growing season metrics and NPP and the impacts of growing season dynamics on NPP in forestland and grassland in the whole China and different regions. We hypothesized that the changes in growing season metrics showed obvious overall consistency and regional differences, and these changes would lead to the overall increase of NPP in forestland and grassland of China.

## Materials and methods

### Data source

The land surface phenology (LSP) products with a spatial resolution of 5 km (0.05-deg), including SOS, EOS and LOS, from the Vegetation Index & Phenology Laboratory, the University of Arizona, were used (https://vip.arizona.edu/viplab_data_explorer.php). These data were derived from Moderate Resolution Imaging Spectroradiometer (MODIS) C5 data during 2000–2016 and Advanced Very High Resolution Radiometer (AVHRR) LTDR v4 data during 1981–1999 using a modified Half-Maximum method [10, 26]. In the LSP products, a pixel reliability (rank) layer was also included, which provided the quality information of remote sensing data of different time series used to derive growing season metrics and to some extent reflected the accuracy of estimation results.

Net primary productivity (NPP) products at 0.05-deg (approximately 5 km) resolution during 1981–2016, estimated by the improved Multisource Data Synergized Quantitative-Net Primary Productivity (MuSyQ-NPP) model [23], were also used. These data came from National Earth System Science Data Center, National Science & Technology Infrastructure of China (http://www.geodata.cn), with a time resolution of 8 days. In the MuSyQ-NPP model, daily gross primary productivity (GPP) was estimated using a light-use efficiency model and expressed as the product of incident photosynthetically active radiation (PAR) and the fraction absorbed by vegetation (FPAR) [23, 27], and the NPP products were based on the Global LAnd Surface Satellite (GLASS) leaf area index and FPAR products, as well as ERA-Interim meteorological data.

Land use and land cover data with 100 m spatial resolution and four periods (1990, 2000, 2010 and 2020) were used to extract the forestland and grassland in China during 1981–2016. These data were retrieved and updated through human-computer interaction methods based on geographic knowledge and remote sensing satellite images such as Landsat TM/ETM and Landsat 8, with reference to the land-use remote sensing mapping system of China [28]. According to the land resources and utilization attributes, the first-level land-use mapping system mainly included six categories, i.e. cropland, forestland, grassland, water area, construction land and unused land. In this study, the areas that had always been forestland or grassland in the above four periods were firstly selected, and the initial areas of forestland and grassland were 178.65 × 10^4^ square kilometers and 210.06 × 10^4^ square kilometers, respectively.

### Methods

#### Extraction and calculation of growing season metrics

**Fig. 1:**
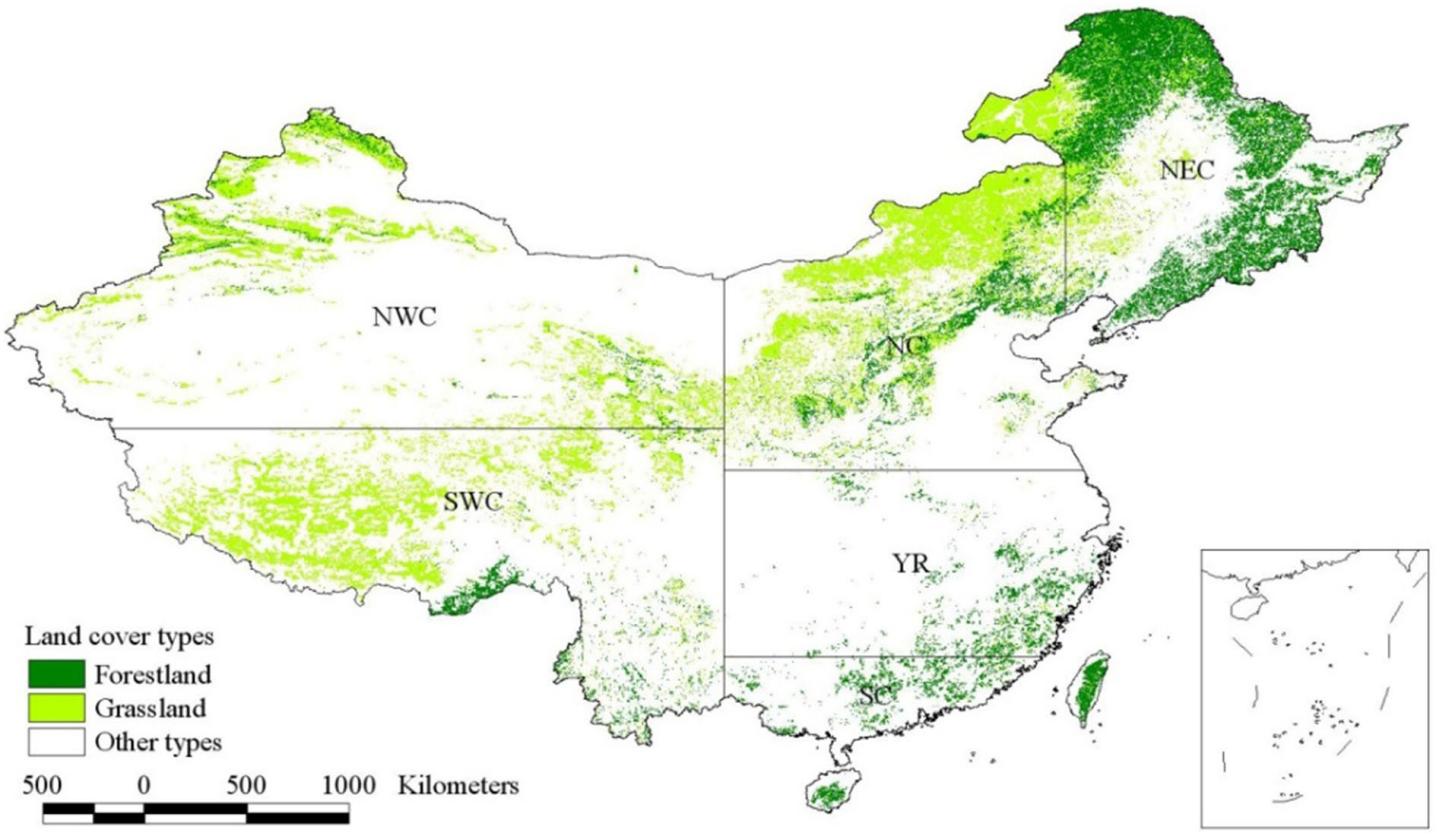
Study areas and the distribution of eventually selected forestland and grassland in China [according to climate, vegetation and other physical geography, the whole China was further divided into six regions: Northwest China (NWC), North China (NC), Northeast China (NEC), Southwest China (SWC), the mid-lower Yangtze River valley (YR) and South China (SC)]

Vegetation indices (VIs), calculated by surface reflectance, are widely used to estimate large-scale LSP, and satellite-derived normalized difference vegetation index (NDVI) and enhanced vegetation index (EVI) are two of the most commonly used VIs. Considering the spatial continuity, the length of data time series, and the ability to truly reflect the dynamics of vegetation growth and development [29, 30], the growing season metrics (SOS, EOS and LOS) retrieved from EVI in LSP products were used here. In the initially selected forestlands and grasslands, pixels with growing season metrics for at least 30 consecutive years between 1981 and 2016, and reliabilities (ranks) of 0–2 for most years, were eventually identified as study areas (Fig. 1). In this paper, the study areas covered 234.06 × 10^4^ square kilometers, including 91.37 × 10^4^ square kilometers of forestland and 142.69 × 10^4^ square kilometers of grassland.

The whole of China was further divided into six regions according to climate, vegetation and other physical geography [11, 31]: Northwest China (NWC), North China (NC), Northeast China (NEC), Southwest China (SWC), the mid-lower Yangtze River valley (YR) and South China (SC), as shown in Fig. 1. Based on the spatial distribution of forestland and grassland in the study areas, the values of annual growing season metrics in forestland and grassland as a whole, and those in forestland and grassland respectively were extracted. According to the number of grids in each region, the arithmetic averages were also calculated to obtain the regional-averaged values of annual growing season metrics.

#### Processing and calculation of annual NPP

The NPP products with 5 km and 8-day resolution were firstly converted from TIFF format to Grid format, and then the annual NPP of each grid was obtained by daily NPP accumulation. According to the distribution of forestland and grassland in the study areas (Fig. 1), annual NPP values in forestland and grassland as a whole, and those in forestland and grassland respectively were also extracted. For analyzing the differences of NPP in six regions of China, the arithmetic average method based on the number of grids was also used to calculate the regional-averaged NPP values.

#### Analysis of changes in growing season metrics and NPP

Based on the annual growing season metrics and NPP values in forestland and grassland in China during 1981–2016, the temporal and spatial changes, including their inter-annual variations and the long-term trends were analyzed at the regional average level and pixel level. The temporal and spatial changes of growing season metrics and NPP in different land cover types (forestland and grassland) and different regions of China were also analyzed.

Many methods are used to detect the changes of time series, among which the least-square fitting of linear trend estimation is widely used [11, 31]. The linear trends of growing season metrics and NPP for each pixel were calculated using the ordinary least-square regression method, and the two-tailed t test was used to calculate the statistical significance. The linear regression coefficient (slope) reflected the trend and magnitude of change. For SOS and EOS, the negative or positive slope indicated the advance or delay of the timing of growing season, respectively, while for LOS, the negative or positive slope reflected the shortening or extension of the length of growing season, respectively. Similarly, the negative or positive slope of NPP indicated the decrease or increase of NPP, respectively.

#### Correlation analysis between growing season metrics and NPP

Correlation coefficients are often used in statistics to determine the relationship between two given variables. The Pearson’s correlation coefficients between growing season metrics and NPP were calculated on a grid scale, and their significances were tested using a two-tailed t test. Similarly, the correlation and significance between regional-averaged growing season metrics and annual NPP in different land cover types and different regions of China were also analyzed. The spatial distributions of correlation coefficients were displayed with ArcGIS 10.3, and the statistical results were displayed using Microsoft Office Excel 2013.

## Results

### Temporal and spatial changes in growing season metrics

#### Inter-annual variations in growing season metrics

**Fig. 2:**
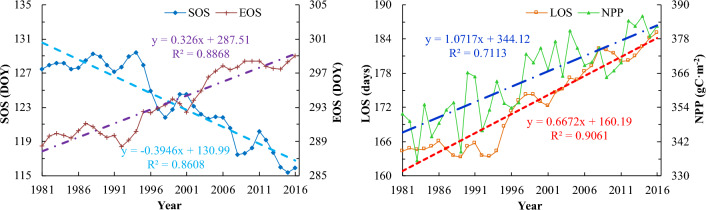
Inter-annual variations of SOS, EOS, LOS and NPP in forestland and grassland in China during 1981–2016 (the light blue, purple, red and blue thick dashed lines are the linear trends estimated by ordinary least-squares regression for SOS, EOS, LOS and NPP, respectively)

The annual variations of SOS, EOS and LOS in forestland and grassland in China were shown in Fig. 2, and all metrics had clear trends during 1981–2016. The SOS, EOS and LOS in the study areas were advanced, delayed and extended, with a rate of 3.9, 3.3 and 6.7 days decade^−1^, respectively, and the trends were all statistically significant at 0.001 level (Table 1). The inter-annual variations of growing season metrics were also different between forestland and grassland. In forestland, the SOS, EOS and LOS were significantly advanced, delayed and extended at a rate of 3.2, 3.4 and 5.2 days decade^−1^, respectively, while those in grassland were significantly advanced, delayed and extended at a rate of 4.4, 3.2 and 7.6 days decade^−1^, respectively (Table 1).

**Table 1:** Trends of SOS, EOS, LOS and NPP and in different land cover types and different regions in China from 1981 to 2016

Land cover types or regions	SOS	EOS	LOS	NPP
Land cover types				
Forestland and Grassland	− 3.946***	3.260***	6.672***	10.717***
Forestland	− 3.231***	3.371***	5.170***	10.328***
Grassland	− 4.403***	3.189***	7.634***	10.966***
Regions				
Northwest China	− 3.877***	4.831***	8.715***	14.739***
North China	− 5.889***	2.948***	8.839***	16.785***
Northeast China	− 0.476	0.675***	1.167***	3.299
Southwest China	− 4.630***	3.684***	7.306***	10.207***
Mid-lower Yangtze River valley	− 7.468***	7.748***	13.095***	18.953***
South China	− 15.224***	17.417***	23.687***	8.311

*Trend is significant at the 0.05 level

**Trend is significant at the 0.01 level

***Trend is significant at the 0.001 level

From the regional average, SOS was advanced, EOS was delayed and LOS was extended in all six regions of China during 1981–2016 (Table 1), and the trends were statistically significant in almost all regions and for all three growing season metrics. Among the six regions of China, South China (SC) had the greatest SOS advance, EOS delay and corresponding LOS extension, followed by the mid-lower Yangtze River valley (YR), while in Northeast China (NEC), SOS, EOS and LOS were the least changed.

#### Spatial trends in annual growing season metrics

**Fig. 3:**
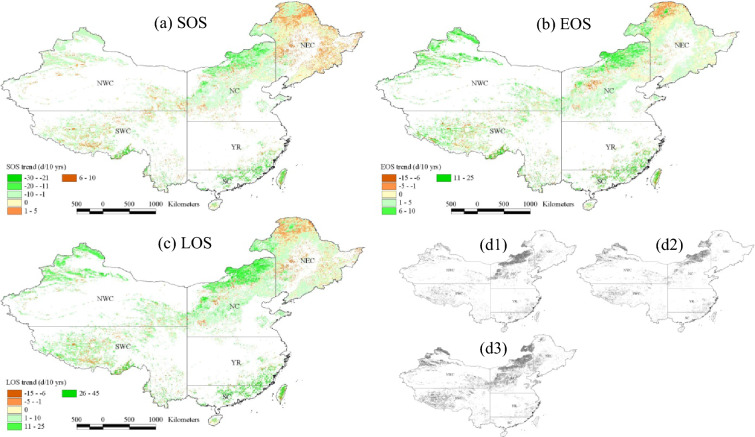
Spatial trends of SOS (**a**), EOS (**b**) and LOS (**c**) in forestland and grassland in China during 1981–2016 (the gray areas in **d1**–**d3** respectively indicate that the trends of SOS, EOS and LOS pass the significance test at the 0.05 level)

During 1981–2016, SOS was advanced in 57.7% of the study areas (Table 2), mainly distributed in the northwestern part of Northwest China (NWC), the northern and western parts of North China (NC), the southwestern part of Southwest China (SWC), northwestern NEC, southeastern YR, and central and eastern SC (Fig. 3a), and the trends were significant in 23.7% of the study areas, with the advancing rates of 1–30 days decade^−1^. The areas with significant delay of SOS accounted for 3.3% of the study areas (Table 2). The EOS was delayed in 65.8% of the study areas, mainly distributed in NWC, NC, NEC, SWC, southeastern YR, and central and eastern SC (Fig. 3b), and the trends were significant in 21.0% of the study areas, where the EOS was delayed mainly at rates of 1–25 days decade^−1^. Over the past 36 years, 73.6% of the study areas had prolonged LOS (Table 2), mainly distributed in NWC, NC, western and eastern NEC, western and southern SWC, southeastern YR, and central and eastern SC (Fig. 3c), and the trends were significant in 40.5% of study areas, with prolonging rates of 1–45 days decade^−1^.

**Table 2:** The area percentage of different trends and significances for growing season metrics and NPP in different land cover types and different regions in China (Units: %)

Land cover types or regions	SOS				EOS				LOS				NPP			
	−		+		−		+		−		+		−		+	
	Total	Sig	Total	Sig	Total	Sig	Total	Sig	Total	Sig	Total	Sig	Total	Sig	Total	Sig
Land cover types																
Forestland and Grassland	57.7	23.7	18.6	3.3	11.0	1.9	65.8	21.0	12.6	4.5	73.6	40.5	22.3	3.0	71.7	19.9
Forestland	39.1	8.9	24.2	3.3	12.3	1.3	54.1	7.6	15.4	3.3	61.7	15.3	21.8	1.6	74.7	16.8
Grassland	69.5	33.2	15.0	3.3	10.1	2.3	73.3	29.5	10.9	5.2	81.2	56.7	22.7	4.0	69.8	21.9
Regions																
Northwest China	73.9	25.0	12.5	1.7	6.0	1.3	84.2	43.1	7.2	3.5	87.8	68.3	13.6	2.4	78.1	18.4
North China	78.3	44.5	8.3	2.5	8.7	2.1	74.1	25.8	6.7	3.8	87.6	56.8	27.7	6.3	67.5	6.6
Northeast China	25.2	5.5	31.1	3.8	10.7	0.5	48.8	6.2	19.1	4.0	52.3	13.6	30.3	1.8	65.4	19.8
Southwest China	65.6	22.6	18.5	3.5	16.2	3.7	67.5	20.5	14.5	6.3	77.2	47.3	5.6	1.7	84.6	49.8
Mid-lower Yangtze River valley	69.0	29.8	17.8	7.1	21.0	7.4	67.1	29.6	15.4	10.6	78.2	31.1	16.6	0.0	81.4	0.4
South China	73.9	21.8	12.7	7.1	20.0	2.7	65.3	13.7	12.2	4.4	75.5	8.9	33.9	1.6	63.3	1.1

−: negative trend; +: positive trend; Sig: trend is significant at the 0.05 level

The proportion of area with significant changes in growing season metrics was larger in grassland than in forestland. In grassland, the areas with significantly advanced SOS, delayed EOS and prolonged LOS accounted for 33.2%, 29.5% and 56.7% of total grassland areas, while those in forestland accounted for 8.9%, 7.6% and 15.3%, respectively (Table 2). Among the six regions of China, NWC and NC showed the most obvious changes in growing season metrics, while NEC showed the least obvious changes. The area proportion of SOS significantly advanced was the largest in NC (44.5%), and those of EOS significantly delayed and LOS significantly prolonged were the largest in NWC (43.1% and 68.3%, respectively).

### Temporal and spatial changes in annual NPP

#### Inter-annual variations in averaged NPP

Annual NPP in forestland and grassland in China was increased significantly at a rate of 10.7 gC m^−2^ decade^−1^ during 1981–2016 (Fig. 2). The increasing trend of NPP was consistent with the lengthening of the growing season. In forestland and grassland, annual NPP was increased significantly at a rate of 10.3 and 11.0 gC m^−2^ decade^−1^, respectively (Table 1), but the inter-annual variation characteristics were different. Forestland NPP was increased slightly during 1981–1990 (r = 0.23, p > 0.05) and then increased significantly at a rate of 22.0 gC m^−2^ decade^−1^ during 1991–2004 (r = 0.66, p < 0.01) (Figure omitted). From 2005 to 2016, forestland NPP was increased slightly at a rate of 3.8 gC m^−2^ decade^−1^ (r = 0.17, p > 0.05). Grassland NPP was changed slightly during 1981–1990 (r = 0.28, p > 0.05) and then increased rapidly at a rate of 12.2 gC m^−2^ decade^−1^ during 1991–2016 (r = 0.74, p < 0.001).

NPP increased in all six regions of China (Table 1). In NWC, NC, SWC and YR, annual NPP in forestland and grassland was increased significantly at a rate of 14.7, 16.8, 10.2 and 19.0 gC m^−2^ decade^−1^, respectively, but in NEC and SC, the increasing trend of NPP was not significant from 1981 to 2016. The inter-annual variation characteristics of NPP in different regions were not completely the same (Figure omitted). In NWC, NC and SWC, annual NPP was continued to increase during 1981–2016, while in YR and SC, NPP was increased before 2004, and then decreased. In NEC, annual NPP was increased before 2000, and then decreased first and then increased.

#### Spatial trends in annual NPP

**Fig. 4:**
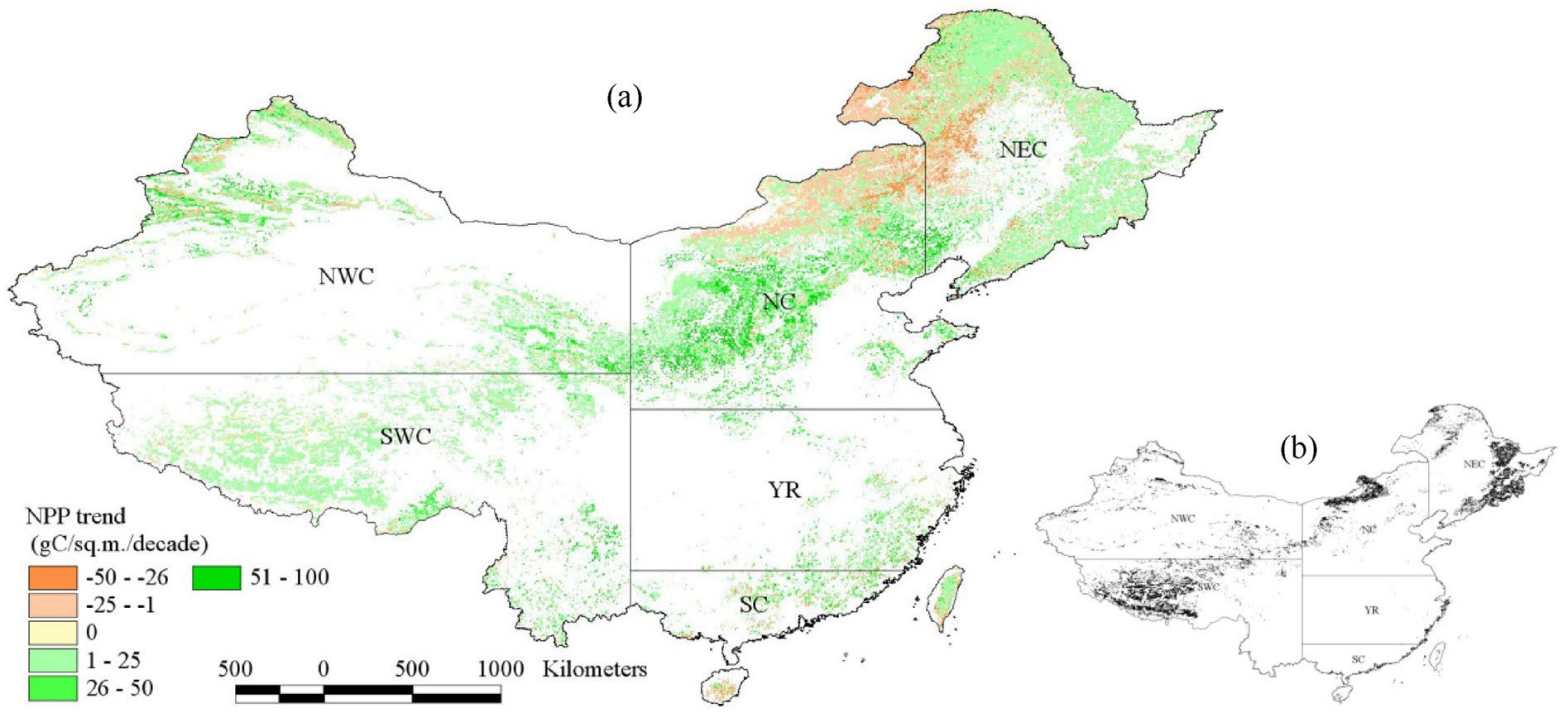
Spatial trends of NPP (**a**) in forestland and grassland in China during 1981–2016 (the gray areas in **b** indicate that the trends pass the significance test at the 0.05 level)

During 1981–2016, NPP was increased at a rate of 1–100 gC m^−2^ decade^−1^ in most areas of forestland and grassland in China (Fig. 4a), especially in Qinghai-Tibetan Plateau, the eastern and northern NEC, western NC and most areas of NWC, the increasing trend was statistically significant (Fig. 4b). In northern NC, northwestern NEC, some areas of NWC, Hainan and Taiwan, NPP was decreased at a rate of 1–50 gC m^−2^ decade^−1^. For the study areas as a whole, the areas where NPP increased were larger than those where NPP decreased, and the areas where NPP increased significantly accounted for 19.9% of the total study areas (Table 2).

In forestland, grassland and different regions of China, the areas where NPP increased were all larger than those where NPP decreased, and the areas where NPP increased significantly were generally larger than those with significant NPP decrease (Table 2). In the forestland, the areas where NPP increased accounted for 74.7% of the total forestland areas, and the areas where NPP increased significantly accounted for 16.8%. Grassland NPP increased in 69.8% of the total grassland areas, and the areas where NPP increased significantly accounted for 21.9%. In six regions of China, the areas where NPP increased were ranged from 63.3 to 84.6% of the regional study areas, while those with NPP decrease were between 5.6% and 33.9% of the regional study areas. In SWC, the proportion of areas where NPP increased significantly was the highest (49.8%).

### The relationships between NPP and growing season metrics

#### The correlation between regional NPP and growing season metrics

From 1981 to 2016, NPP was negatively correlated with SOS and positively correlated with EOS and LOS, and all passed the significance test at the 0.001 level (Table 3). In forestland and grassland respectively, NPP was also negatively correlated with SOS and positively correlated with EOS and LOS, and the correlations were also statistically significant, indicating that earlier spring, later autumn and longer growing season can all promote the increase of annual NPP. The correlation between NPP and growing season metrics was significant in most regions of China, especially in NWC, NC and SWC, the correlation was statistically significant at the 0.001 level (2-tailed). In NEC, the correlation between NPP and three growing season metrics was not significant. In comparison, the correlation between NPP and growing season metrics was the best in SWC, followed by NWC and NC, and the worst in NEC.

**Table 3:** The correlation coefficient between NPP and SOS, EOS and LOS in different land cover types and different regions in China from 1981 to 2016

Land cover types or regions		SOS	EOS	LOS
Land cover types	Forestland and Grassland	− 0.790**	0.781**	0.814**
	Forestland	− 0.622**	0.494*	0.656**
	Grassland	− 0.714***	0.691***	0.721***
Regions	Northwest China	− 0.758***	0.804***	0.795***
	North China	− 0.737***	0.647***	0.725***
	Northeast China	− 0.181	0.199	0.236
	Southwest China	− 0.811***	0.802***	0.813***
	Mid-lower Yangtze River valley	− 0.414**	0.460**	0.457**
	South China	− 0.303	0.152	0.342*

*Correlation is significant at the 0.05 level

**Correlation is significant at the 0.01 level

***Correlation is significant at the 0.001 level

#### Spatial differences in correlation between NPP and growing season metrics

**Fig. 5:**
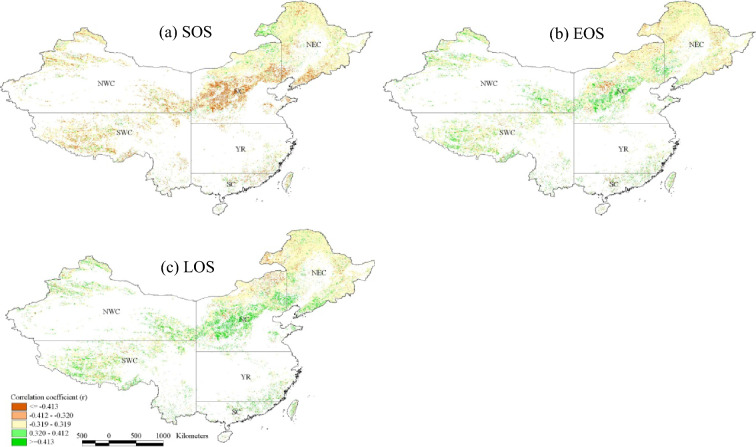
Pearson’s correlation coefficients (r) between NPP and SOS (**a**), EOS (**b**) and LOS (**c**) in forestland and grassland in China during 1981–2016 (r > 0.319: correlation is significant at the 0.05 level; r > 0.412: correlation is significant at the 0.01 level)

In the past 36 years, there was a significant negative correlation between NPP and SOS in 22.0% of the study areas and a significant positive correlation in 6.3% of the study areas (Table 4). The areas of significant correlation were mainly distributed in northwestern and southeastern NWC, northern and central NC, NEC and western SWC (Fig. 5a). In 7.5% of the study areas, there was a significant negative correlation between NPP and EOS, and in 16.3% of the study areas, NPP was significantly and positively correlated with EOS. Areas of significant correlation were mainly located in northwestern and southeastern NWC, northern and central NC, northern and western NEC and western SWC (Fig. 5b). NPP showed a significant negative correlation with LOS in 7.1% of the study areas, and a significant positive correlation in 22.8% of the study areas. The significant correlation areas were mainly distributed in northwestern and southeastern NWC, northeastern and central NC, western and southern NEC and western SWC (Fig. 5c). The areas with significant positive correlation between NPP and LOS were larger than those between NPP and EOS.

**Table 4:** The area percentage of NPP significantly correlated with growing season metrics (p < 0.05) in different land cover types and different regions in China during 1981–2016 (units: %)

Land cover types or regions	SOS		EOS		LOS	
	−	+	−	+	−	+
Land cover types						
Forestland and Grassland	21.95	6.29	7.53	16.25	7.07	22.83
Forestland	20.64	5.60	8.28	13.54	6.83	20.17
Grassland	22.75	6.71	7.07	17.90	7.23	24.56
Regions						
Northwest China	22.18	5.53	3.60	24.52	4.97	27.28
North China	31.38	5.92	8.52	19.65	7.80	32.16
Northeast China	11.36	6.04	7.27	5.90	6.99	10.13
Southwest China	25.82	6.15	7.43	20.82	5.95	27.01
Mid-lower Yangtze River valley	22.28	10.61	11.30	20.95	11.44	30.37
South China	39.53	19.60	28.80	38.35	22.61	51.11

− negative correlation; + positive correlation

The proportion of areas where NPP was significantly correlated with growing season metrics was slightly higher in grassland than in forestland (Table 4). In forestland, there were 20.6%, 13.5% and 20.2% of the total forestland areas where NPP was significantly correlated with advanced SOS, delayed EOS and extended LOS, respectively. In grassland, the corresponding proportion of areas was 22.8%, 17.9% and 24.6%, respectively. Among the six regions of China, SC had the highest proportion of areas where NPP was significantly correlated with growing season metrics (Table 4). There were 39.5%, 38.4% and 51.1% of the regional study areas where NPP was significantly correlated with SOS, EOS and LOS, respectively. NEC had the lowest proportion of areas where NPP was significantly correlated with SOS, EOS and LOS, with the values of 11.4%, 5.9%, and 10.1% of the regional study areas respectively.

## Discussion

### The change in growing season metrics

In the last few decades, much of the northern hemisphere has experienced significantly advanced onset, delayed end and extended length of growing season from climate warming based on satellite images, model simulations and in-situ observations [9, 11]. In the forestland and grassland in China, advanced SOS, delayed EOS and extended LOS occurred in most of the study areas (Fig. 3), especially in grassland, the areas with significantly advanced SOS, delayed EOS and prolonged LOS accounted for 33.2%, 29.5% and 56.7% of total grassland areas, respectively (Table 2). The proportion of areas where growing season metrics changed significantly obtained in our study is close to some existing regional studies. For example, in the temperate grassland and desert zones of China, 68% of natural vegetation pixels showed an advancing trend in SOS, of which 37% of pixels advanced significantly [22]. In the arid and semi-arid areas of northern China, advanced SOS, delayed EOS and extended LOS accounted for 74.2%, 78.4% and 83.4% of the total vegetation areas, respectively, and the trends were significant in 44.3%, 40.6% and 52.6% of vegetated areas, respectively [13].

The SOS, EOS and LOS were advanced, delayed and extended respectively at a rate of 3.9, 3.3 and 6.7 days decade^−1^ during 1981–2016, and the trends in grassland were slightly higher than those in forestland (Fig. 2; Table 1). Our results are consistent with existing studies in some regions of China. For example, in the Inner Mongolia grassland, SOS was advanced at 4.8 days decade^−1^ and EOS was delayed at 4.2 days decade^−1^, resulting in LOS extending at 9.1 days decade^−1^ between 2002 and 2014 [12]. During 2001 to 2016, SOS was significantly advanced by 5.2 days decade^−1^ throughout the freshwater marshes of Northeast China [32]. However, there are also studies with trend values lower than our results. For instance, Li et al. [22] showed that in the temperate grasslands and deserts of China, the SOS was significantly advanced at a rate of 1.4 days decade^−1^ from 1982 to 2015. Ma et al. [8] found that the EOS was delayed by 1.62 days decade^−1^ across temperate grasslands of China during 1982–2015. This has much to do with the difference in research scope, research period, data sources, data processing methods and estimation model selection [13, 15].

### The change of annual NPP

NPP plays an important role in evaluating ecological carrying capacity and understanding the global carbon cycle [20, 33]. From 1981 to 2016, NPP increased in most study areas, especially in Qinghai-Tibetan Plateau, the eastern and northern NEC, western NC and most areas of NWC, the increasing trend was statistically significant (Fig. 4). In northern NC, northwestern NEC, some areas of NWC, Hainan and Taiwan, NPP decreased. The variation pattern of NPP obtained in this study is also in line with the results of other studies. For example, Liu et al. [20] showed that NPP in China increased in northwest and central Inner Mongolia, the Tibetan Plateau and the coastal areas of southeastern China, but in NC, Changbai mountains and the lower reaches of Yangtze River, it decreased during 2001–2014. In the forestland and grassland in China, the areas where NPP increased were much larger than those of NPP decrease (Table 2). Liu et al. [7] also showed that NPP in 66.34% of grassland area in China exhibited an increasing trend from 1982 to 2016, and the areas with significant increase were mainly distributed in Tibetan Plateau and the northern part of Xinjiang.

Annual NPP in the entire study areas increased at a rate of 10.7 gC m^−2^ decade^−1^ during 1981–2016, and the increasing rate of NPP was 10.3 and 11.0 gC m^−2^ decade^−1^ in forestland and grassland, respectively (Fig. 2). Our results are similar to those of Shen et al. [5], which showed that the annual average NPP increased significantly from 2000 to 2020 by 11.70 ± 1.07 gC m^−2^ decade^−1^ in marshes of the Qinghai-Tibet Plateau, but lower than those from Ma et al. [4], which indicated that during 2000–2020, the annual NPP of temperate grasslands in China increased significantly at a rate of 4.0 gC m^−2^ yr^−1^ with the largest increase in temperate meadow (5.4 gC m^−2^ yr^−1^) and the smallest increase in temperate desert steppe (2.2 gC m^−2^ yr^−1^). Although the results are inconsistent due to differences in study scope, study time, data sources, etc., most studies show an overall increase in NPP in China in recent decades. For example, Liang et al. [33] found that NPP in China exhibited a significant upward trend at both the national level and the biome level from 1982 to 2010, and the annual increase of NPP was 0.011 Pg C or 0.42%. Zhao et al. [34] showed that the forestland NPP in China had a fluctuating growth trend from 1982 to 2019, with obvious inter-annual fluctuation.

### The relationship between NPP and growing season metrics

Phenology and productivity are key parameters of ecosystems, and phenology plays an important role in dynamic evaluation of plant productivity [24]. From 1981 to 2016, NPP was negatively correlated with SOS and positively correlated with EOS and LOS (Fig. 5), and the areas with significant correlation accounted for 22.0%, 16.3% and 22.8% of the study areas, respectively (Table 4). In other words, advanced SOS, delayed EOS and extended LOS can promote the increase of NPP. The strong relationship between growing season metrics and NPP was also found by other scholars. For example, Piao et al. [9] showed that LOS was strongly correlated with NPP, and annual NPP increased by 2.8 gC m^−2^ for each day of LOS extension across the Northern Hemisphere. In the grassland and forestland in Hebei Province, SOS was negatively correlated with NPP, while LOS and EOS were positively correlated with NPP [35]. On the Mongolian Plateau, SOS and EOS contributed significantly to spring NPP and autumn NPP, respectively, namely, SOS had a significant negative correlation with spring NPP, EOS had a significant positive correlation with autumn NPP, and the lengthening of LOS led to the increase of annual NPP during 1982–2011 [24].

However, the relationship between plant phenology and NPP remains largely uncertain due to various disturbance mechanisms [24]. For example, in Inner Mongolia, SOS showed a significant advancing trend, but spring NPP did not increase significantly, thus there was no significant negative correlation between SOS and NPP in spring during 2000–2017 [15]. In the northern Tibetan Plateau, NPP increased and EOS showed an advancing trend owing to increased seasonal precipitation, so EOS exhibited negative correlation with NPP during 2000–2020 [16]. Zhang et al. [36] showed that SOS had the strongest positive correlation with NPP, i.e. a delayed SOS generally caused an incremental NPP in the current year, and vice versa, but the relationships between EOS, LOS and annual NPP were not as obvious as SOS in the temperate grasslands of China. Wu et al. [37] also found that the SOS was correlated positively with precipitation and NPP, while the EOS and LOS were negatively with precipitation and NPP in arid Central Asia. In this study, the correlation between NPP and growing season metrics also had obvious regional difference, higher in NWC, NC and SWC and lower in NEC and SC (Table 3).The study area, data source, analysis method, vegetation type, hydrothermal pattern, community structure and other factors may cause the difference in response of NPP to phenological changes [17].

NPP is the difference between carbon absorbed by photosynthesis and carbon released by autotrophic respiration [38], which reflects the absorption rate of atmospheric carbon by vegetation [39]. Over the last few decades, global warming has led to longer growing seasons in earlier spring and later fall, and plants have more days for photosynthesis and accumulating biomass [14, 40], which may increase productivity. However, the relationship between growth season length and productivity is not necessarily linear [3], and the physiological processes of vegetation are affected by changes in hydrothermal conditions [37]. Meteorological factors, including temperature, precipitation and solar radiation, have obvious effects on phenology and NPP [36], and temperature and precipitation play different roles in the diverse responses of NPP to phenological dynamics [18]. In the Tibetan Plateau, the extension of LOS could contribute to the increasing of NPP, but this correlation had regional differences because of the changing dominant meteorological factors [21], and temperature and precipitation had different effects on the relationship between NPP and phenology under different climatic regimes [18]. Meteorological factors also had different roles in the response of NPP to phenological changes in different temperature and precipitation zones of the Loess Plateau, with distinct spatial changes [14].

It is generally believed that the increase of temperature in spring before SOS can lead to the effective accumulated temperature reaching seed germination and leaf unfolding earlier [41]. Moreover, photosynthetic absorption is generally limited by temperature, and rising temperature can accelerate the growth and greenness of vegetation [42]. Plants green earlier in a warmer spring and the increase in productivity caused by the enhancement of photosynthesis is greater than the consumption from respiration, leading to an increase in NPP [21, 40]. Previous studies have also shown that a certain amount of spring precipitation is a trigger for the spring green-up of temperate grasslands in China [6]. Under the condition that the heat required for plant growth is satisfied, the increase of precipitation in summer and autumn can improve the supply of soil water to vegetation, promote the increase of photosynthetic rate and delay the vegetation entering the withered stage, thus improve the productivity of desert steppe in Inner Mongolia [15]. In the arid northwestern region of Loess Plateau, China, the increase in temperature caused a decrease in NPP due to the lengthening of the growing season, but the increase in precipitation delayed the dormancy of plants, allowing more time for photosynthesis and increasing the autumn NPP [14].

Many other factors also affect the relationship between NPP and growing season in forestland and grassland, such as geographical location [17], topographic conditions [18, 21] and vegetation types [16, 17]. In the humid southeast parts of the Loess Plateau, China, advanced SOS and delayed EOS significantly caused the increasing of spring NPP and autumn NPP, respectively, but in the arid northwest regions, spring and autumn NPP did not increase significantly [14]. Wang et al. [18] showed that the response pattern of NPP to growing season changes was mainly controlled by local climatic conditions and topographic characteristics in Tibetan Plateau. Qiu et al. [17] noted that the responses of NPP to growing season metrics showed obvious differences and variations in vegetation type and geographical location, namely, NPP was positively correlated with LOS and negatively correlated with SOS in shrubland, but in deciduous coniferous forest, deciduous broadleaf forest and meadow the response was different due to the geographical location. The area proportion of NPP significantly correlated with growing season was higher in grassland than in forestland in China (Table 4). Thus, the influence of growing season on NPP is complex, and various environmental factors should be further considered in future studies [8, 35].

### Uncertainties and limitations

It is worth noting that in this study, there are some uncertainties in the datasets and methods used to estimate the growing season metrics, NPP, and to determine the relationship between them. Firstly, satellite-based techniques have been widely used for phenological monitoring and NPP estimation. However, the influence of atmosphere, clouds, and solar angle on satellite remote sensing data may lead to uncertainties in LSP products and NPP products [43], and the spatiotemporal diversities and ecological complexities of vegetation biochemical processes also bring some uncertainties to the retrieval of vegetation ecological parameters by remote sensing [18]. Secondly, this study used four periods of land use and land cover data to extract the unchanged forestland and grassland as the study objects, and the effect of human activities on forestland and grassland may not be completely excluded [8]. Thus the uncertainties also arise from the land use and land cover data as each pixel may not reflect the actual land cover type within a 100 m × 100 m area [44]. Finally, although the applicability and reliability of MuSyQ-NPP model in estimating global NPP has been proved [23, 27], there are still some uncertainties in its application in China, which have a certain impact on the current research results, and further research on the comparison and mutual validation of multi-source NPP data is needed. Given the above uncertainties and limitations, it is necessary to explore higher quality datasets and more reliable methods to characterize the temporal and spatial variations of phenology and NPP, and to identify their relationships and the influencing mechanisms more accurately [16].

## Conclusions

From 1981 to 2016, SOS was advanced, EOS was delayed, LOS was prolonged and NPP was increased in the forestland and grassland of China as a whole, and the advanced SOS, delayed EOS and extended LOS can promote the increase of annual NPP. In forestland, grassland and different regions of China, the changes in growing season metrics and NPP and their relationships are somewhat different. The proportion of area with significant change of growing season metrics and NPP and the proportion of area with significant correlation were greater in grassland than in forestland. In Northwest China and North China, the changes in growing season metrics were obvious and the correlation between NPP and growing season metrics was strong among the six regions of China.

Our results highlight the overall consistent spatial and temporal changes and correlations between growing season metrics and NPP in China, but there are some distinctions in different land cover types and different regions of China. Influenced by many factors, the changes of growing season metrics and NPP and their mutual relationship are complicated and remain largely uncertain. With global warming and regional ecological environment changes, the influence of multiple factors such as geographical location, dominant meteorological factors, topographic conditions, vegetation types and even human activities should be adequately considered in further studies, combined with higher quality datasets and more reliable methods to reduce the uncertainties.

## Data Availability

The datasets during and/or analysed during the current study available from the corresponding author on reasonable request.

## References

[1] IPCC (2021). Climate change 2021: the physical science basis. contribution of working group I to the sixth assessment report of the intergovernmental panel on climate change.

[2] Zhang Y, Yang P, Gao Y, Leung RL, Bell ML (2020). Health and economic impacts of air pollution induced by weather extremes over the continental U.S. Environ Int.

[3] Richardson AD, Keenan TF, Migliavacca M, Ryu Y, Sonnentag O, Toomey M (2013). Climate change, phenology, and phenological control of vegetation feedbacks to the climate system. Agric Meteorol.

[4] Ma R, Xia C, Liu Y, Wang Y, Zhang J, Shen X (2022). Spatiotemporal change of net primary productivity and its response to climate change in temperate grasslands of China. Front Plant Sci.

[5] Shen X, Liu Y, Zhang J, Wang Y, Ma R, Liu B (2022). Asymmetric impacts of diurnal warming on vegetation carbon sequestration of marshes in the Qinghai Tibet Plateau. Glob Biogeochem Cycles.

[6] Wang G, Huang Y, Wei Y, Zhang W, Li T, Zhang Q (2019). Inner Mongolian grassland plant phenological changes and their climatic drivers. Sci Total Environ.

[7] Liu Y, Zhou R, Ren H, Zhang W, Zhang Z, Zhang Z (2021). Evaluating the dynamics of grassland net primary productivity in response to climate change in China. Glob Ecol Conserv.

[8] Ma R, Shen X, Zhang J, Xia C, Liu Y, Wu L (2022). Variation of vegetation autumn phenology and its climatic drivers in temperate grasslands of China. Int J Appl Earth Obs.

[9] Piao S, Friedlingstein P, Ciais P, Viovy N, Demarty J (2007). Growing season extension and its impact on terrestrial carbon cycle in the Northern Hemisphere over the past 2 decades. Glob Biogeochem Cycles.

[10] White MA, de Beurs KM, Didan K, Inouye DW, Richardson AD, Jensen OP (2009). Intercomparison, interpretation, and assessment of spring phenology in North America estimated from remote sensing for 1982–2006. Glob Change Biol.

[11] Cui L, Shi J, Ma Y (2018). Temporal and spatial variations of the thermal growing season in China during 1961–2015. Meteorol Appl.

[12] Gong Z, Kawamura K, Ishikawa N, Goto M, Wulan T, Alateng D (2015). MODIS normalized difference vegetation index (NDVI) and vegetation phenology dynamics in the Inner Mongolia grassland. Solid Earth.

[13] Cui L, Shi J (2021). Evaluation and comparison of growing season metrics in arid and semi-arid areas of northern China under climate change. Ecol Indic.

[14] Han H, Bai J, Ma G, Yan J, Wang X, Ta Z (2022). Seasonal responses of net primary productivity of vegetation to phenological dynamics in the Loess Plateau, China. Chin Geogr Sci.

[15] Dong X, Yao H, Dai J, Zhu M (2020). Phenological changes of desert steppe vegetation and its effect on net primary productivity in Inner Mongolia from 2000 to 2017. Prog Geogr.

[16] Li X, Zhao C, Kang M, Ma M (2022). Responses of net primary productivity to phenological dynamics based on a data fusion algorithm in the northern Qinghai-Tibet Plateau. Ecol Indic.

[17] Qiu Y, Fan D, Zhao X, Sun W (2017). Spatiotemporal changes of NPP and its responses to phenology in Northeast China. Geogr Geo Inf Sci.

[18] Wang S, Zhang B, Yang Q, Chen G, Yang B, Lu L (2017). Responses of net primary productivity to phenological dynamics in the Tibetan Plateau, China. Agric For Meteorol.

[19] Shi J, Cui L, Tian Z (2020). Spatial and temporal distribution and trend in flood and drought disasters in East China. Environ Res.

[20] Liu G, Sun R, Xiao Z, Cui T (2017). Analysis of spatial and temporal variation of net primary productivity and climate controls in China from 2001 to 2014. Acta Ecol Sin.

[21] Yang B, Wang S, Chang Q, Sun Y, Yin H, Wang X (2015). Response of NPP to phenology changes in the Tibet plateau. Geogr Geo Inf Sci.

[22] Li Y, Zhang Y, Gu F, Liu S (2019). Changes of spring phenology and sensitivity analysis in temperate grassland and desert zones of China. For Res.

[23] Cui T, Wang Y, Sun R, Qiao C, Fan W, Jiang G (2016). Estimating vegetation primary production in the Heihe River Basin of China with multi-source and multi-scale data. PLoS ONE.

[24] Bao G, Chen J, Chopping M, Bao Y, Bayarsaikhan S, Dorjsuren A (2019). Dynamics of net primary productivity on the Mongolian Plateau: joint regulations of phenology and drought. Int J Appl Earth Obs.

[25] Zhu J, Tian Y, Li Q, Liu H, Guo X, Tian H (2023). The current and potential carbon sink in forest ecosystems in China. Acta Ecol Sin.

[26] White MA, Thomton PE, Running SW (1997). A continental phenology model for monitoring vegetation responses to interannual climatic variability. Glob Biogeochem Cycles.

[27] Yu T, Sun R, Xiao Z, Zhang Q, Liu G, Cui T (2018). Estimation of global vegetation productivity from global land surface satellite data. Remote Sens.

[28] Liu J, Kuang W, Zhang Z, Xu X, Qin Y, Ning J (2014). Spatiaotemporal characteristics, patterns and causes of land-use changes in China since the late 1980s. J Geogr Sci.

[29] Cao R, Chen J, Shen M, Tang Y (2015). An improved logistic method for detecting spring vegetation phenology in grasslands from MODIS EVI time-series data. Agric For Meteorol.

[30] Peng D, Wu C, Li C, Zhang X, Liu Z, Ye H (2017). Spring green-up phenology products derived from MODIS NDVI and EVI: intercomparison, interpretation and validation using national phenology network and AmeriFlux observations. Ecol Indic.

[31] Shi J, Cui L (2021). Comparison of seasonal climate in China during the cold and warm phases of ENSO. Clim Res.

[32] Shen X, Liu B, Xue Z, Jiang M, Lu X, Zhang Q (2019). Spatiotemporal variation in vegetation spring phenology and its response to climate change in freshwater marshes of Northeast China. Sci Total Environ.

[33] Liang W, Yang Y, Fan D, Guan H, Zhang T, Long D (2015). Analysis of spatial and temporal patterns of net primary production and their climate controls in China from 1982 to 2010. Agric For Meteorol.

[34] Zhao J, Liu D, Cao Y, Zhang L, Peng H, Wang K (2022). An integrated remote sensing and model approach for assessing forest carbon fluxes in China. Sci Total Environ.

[35] Zhang C, Yuan J (2022). Phenological period of grassland and woodland in Hebei Province and correlation analysis with net primary productivity (NPP) based on MODIS data. Remote Sens Technol Appl.

[36] Zhang C, Zhang Y, Wang Z, Li J, Odeh I (2019). Monitoring phenology in the temperate grasslands of China from 1982 to 2015 and its relation to net primary productivity. Sustainability.

[37] Wu L, Ma X, Dou X, Zhu J, Zhao C (2021). Impacts of climate change on vegetation phenology and net primary productivity in arid Central Asia. Sci Total Environ.

[38] Lieth H (1975). Modeling the primary productivity of the world. Primary productivity of the biosphere.

[39] Ruimy A, Saugier B, Dedieu G (1994). Methodology for the estimation of terrestrial net primary production from remotely sensed data. J Geophys Res-Atmos.

[40] Richardson AD, Black AT, Ciais P, Delbart N, Friedl MA, Nadine G (2010). Influence of spring and autumn phenological transitions on forest ecosystem productivity. Philos Trans R Soc B.

[41] Shi P, Chen Z, Reddy GVP, Hui C, Huang J, Xiao M (2017). Timing of cherry tree blooming: contrasting effects of rising winter low temperatures and early spring temperatures. Agric For Meteorol.

[42] Tanja S, Berninger F, Vesala T, Markkanen T, Hari P, Mäkelä A (2003). Air temperature triggers the recovery of evergreen boreal forest photosynthesis in spring. Glob Change Biol.

[43] Shen X, Liu B, Henderson M, Wang L, Jiang M, Lu X (2022). Vegetation greening, extended growing seasons, and temperature feedbacks in warming temperate grasslands of China. J Clim.

[44] Shen X, Liu B, Jiang M, Lu X (2020). Marshland loss warms local land surface temperature in China. Geophys Res Lett.

